# The impact of COVID-19 on healthcare delivery for people who use opioids: a scoping review

**DOI:** 10.1186/s13011-021-00395-6

**Published:** 2021-08-09

**Authors:** Karen Alexander, Monika Pogorzelska-Maziarz, Angela Gerolamo, Nadia Hassen, Erin L. Kelly, Kristin L. Rising

**Affiliations:** 1 Jefferson College of Nursing, Thomas Jefferson University, 901 Walnut St., Suite 800, Philadelphia, PA 19107 USA; 2 Department of Family and Community Medicine, Sidney Kimmel Medical College, Thomas Jefferson University, 1015 Walnut Street, Suite 40, Philadelphia, PA 19107 USA; 3 Department of Emergency Medicine, Sidney Kimmel Medical College, Thomas Jefferson University, 1020 Sansom Street, Suite 239, Philadelphia, PA 19107 USA

**Keywords:** Opioid use, Healthcare delivery, Pandemic

## Abstract

**Research objective:**

The COVID-19 pandemic disrupted healthcare delivery worldwide with likely negative effects on people who use opioids (PWUO). This scoping review of the original research literature describes the impact of the COVID-19 pandemic on healthcare delivery for PWUO and identifies gaps in the literature.

**Methods:**

This scoping review of the original research literature maps the available knowledge regarding the impact of the COVID-19 pandemic on healthcare delivery for PWUO. We utilized the methodology developed by the Joanna Briggs Institute for scoping reviews, and content analyses methodology to characterize the current state of the literature.

**Results:**

Of the 14 included studies, administrative database (*n* = 11), cross-sectional (*n* = 1) or qualitative (*n* = 2) studies demonstrated service gaps (*n* = 7), patient/provider experiences (*n* = 3), and patient outcomes for PWUO (*n* = 4). In March 2020, healthcare utilization dropped quickly, sharply increasing only for reasons of opioid overdose by May 2020. Service gaps existed in accessing treatment for new patients during the pandemic due to capacity and infrastructure limits. Physicians reported difficulty referring patients to begin an outpatient opioid treatment program due to increased restrictions in capacity and infrastructure. Patients also reported uncertainty about accessing outpatient treatment, but that telehealth initiation of buprenorphine increased access to treatment from home. Disproportionate increases in overdose rates among African Americans were reported in two studies, with differences by race and gender not examined in most studies. Fatal overdoses increased 60% in African Americans during the pandemic, while fatal overdoses in Non-Hispanic White individuals decreased.

**Conclusions:**

In summary, this beginning evidence demonstrates that despite early reluctance to use the healthcare system, opioid overdose-related use of healthcare increased throughout the pandemic. Service delivery for medications to treat OUD remained at or above pre-pandemic levels, indicating the ability of telehealth to meet demand. Yet, racial disparities that existed pre-pandemic for PWUO are intensifying, and targeted intervention for high-risk groups is warranted to prevent further mortality. As the pandemic progresses, future research must focus on identifying and supporting subgroups of PWUO who are at heightened risk for experiencing negative outcomes and lack of access to care.

## Background

Over 58 million people used opioids in 2019, and 500,000 drug-related overdoses occurred, mostly attributable to opioids [1]. Recent work has demonstrated disparities in the rate of opioid-related overdoses in the United States (US) among certain subgroups of the population [2]. Although non-Hispanic white men comprise the largest percentage of overdose deaths, fatal overdoses are increasing at a significantly higher rate among racial minority groups as compared to white men [3]. Geographically, the northeastern US is seeing the highest increase in overdose fatalities with an average increase of 200% in the mid-Atlantic and New England states from 1999 to 2017 [3].

The delivery of quality and timely healthcare to people who use opioids (PWUO) can prevent opioid related mortality and morbidity through harm reduction strategies and medications to treat opioid use disorder (OUD) [4]. Methadone is the most frequently prescribed medication for OUD (MOUD) and is administered daily in an outpatient setting, but with significant thresholds for entering and maintaining treatment, such as waiting lists, frequent urine toxicology, and timed dosing of medication dispensing [5]. Buprenorphine is the second most used MOUD and can be prescribed in an emergency department (ED), Office-based Opioid Treatment (OBOT) or an outpatient Opioid Treatment Program (OTP) [6]. Alongside pharmacologic treatments, psychotherapy, housing support and job training are highly recommended for people in recovery [4].

The care received in these differing settings is influenced greatly by federal and local policies, which have improved greatly in recent years. For example, The SUPPORT Act of 2018 requires OBOT and OTP to be covered by Medicaid and Medicare andthe Comprehensive Addiction and Recovery Act in 2016 made provisions for increased numbers of healthcare providers (including physician’s assistants, and nurse practitioners) to prescribe buprenorphin [7]. The result is an increasing number of patients enrolled in treatment in the last 5 years, especially in rural areas [8, 9].

Until recently, healthcare providers were required to perform in-person physicals prior to prescribing any MOUD under the Ryan Haight Act [7]. The Ryan Haight Act was established to prevent illicit online pharmacies from adding to the opioid epidemic. Federal regulations also placed strict dosing limits on MOUD at OTP with requirements for daily or weekly follow-up visits in early stages of treatment [10]. However, these guidelines surrounding the treatment of OUD were altered during the coronavirus-2019 (COVID-19) pandemic.

**Fig. 1:**
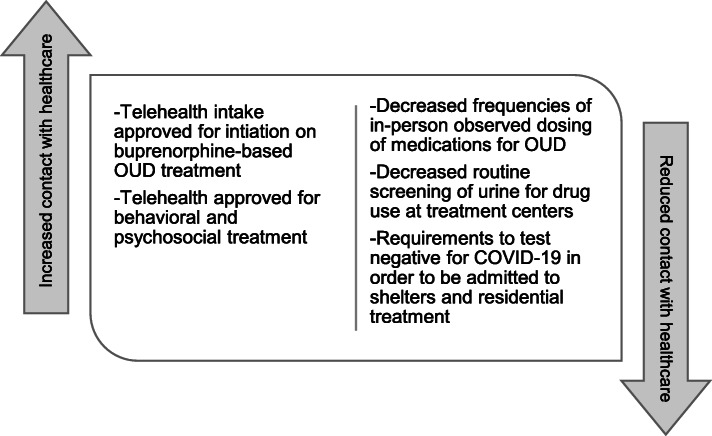
SAMHSA guidance affecting healthcare delivery for PWUO after March 16th, 2020

Due to the nature of the COVID-19 public health crisis, stay-at-home orders were in place globally by March 2020. PWUO then faced restrictions to in-person treatment intake, transportation problems for attending their daily dosing appointments, and technology driven limitations in accessing psychotherapy sessions. Fortunately, federal guidance released on March 16th, 2020 by the Substance Abuse and Mental Health Association (SAMHSA) allowed providers to prescribe buprenorphine without an in-person physical. Barriers to telehealth prescribing of buprenorphine were suspended under a provision within the Ryan Haight Act for public health emergencies. In addition, while methadone induction still required in-person intake appointments, methadone could be prescribed in 14- and 28- day take-home options for patients demonstrating adequate adherence to treatment. The policy changes to health care delivery for PWUO during the COVID-19 pandemic are summarized in Fig. 1.

It is evident from prior work that people who use substances are at greater risk of COVID-19 related disease [11], and that substance use overall has increased substantially during the pandemic [12]. PWUO face increased complexity in managing their health which may have exacerbated their existing marginalization [13]. While previous reviews have examined the intersection of substance use and COVID-19, there is a gap in evidence regarding changes in healthcare delivery for PWUO during the pandemic.

As the pandemic continues, it is important to explore how healthcare delivery can best support the provision of safe and effective care for PWUO. To fill this gap, we conducted a scoping review to assess the answer to this question: What evidence exists regarding the intersection of the COVID-19 pandemic and health care delivery for PWUO?

## Methods

### Data sources and search strategy

Before the study began, we determined the objective of the study, the research question, inclusion criteria, and method. With the assistance of a medical librarian, the database search included English language articles within 3 databases using a defined search strategy. PubMed, the Cumulative Index for Nursing and Allied Health Literature (CINAHL), and OVID Medline were searched, without restricting the date of publication using the terms: (Coronavirus OR COVID) AND (Opioid OR methadone OR Buprenorphine OR suboxone OR opiate) AND (outcome OR overdose OR emergency OR health). No date restrictions were placed as it was presumed all studies that were published using the terms COVID or Coronavirus would be published after January 2020. The search term list was compiled by study team members (K.A., N.H). The five-stage process outlined by Arksey and O’Malley of the Joanna Briggs Institute was used to conduct the scoping review: 1) identify the research question 2) identify the relevant studies 3) study selection 4) chart the data and 5) organize the results [14].

### Screening strategy and inclusion criteria

**Fig. 2:**
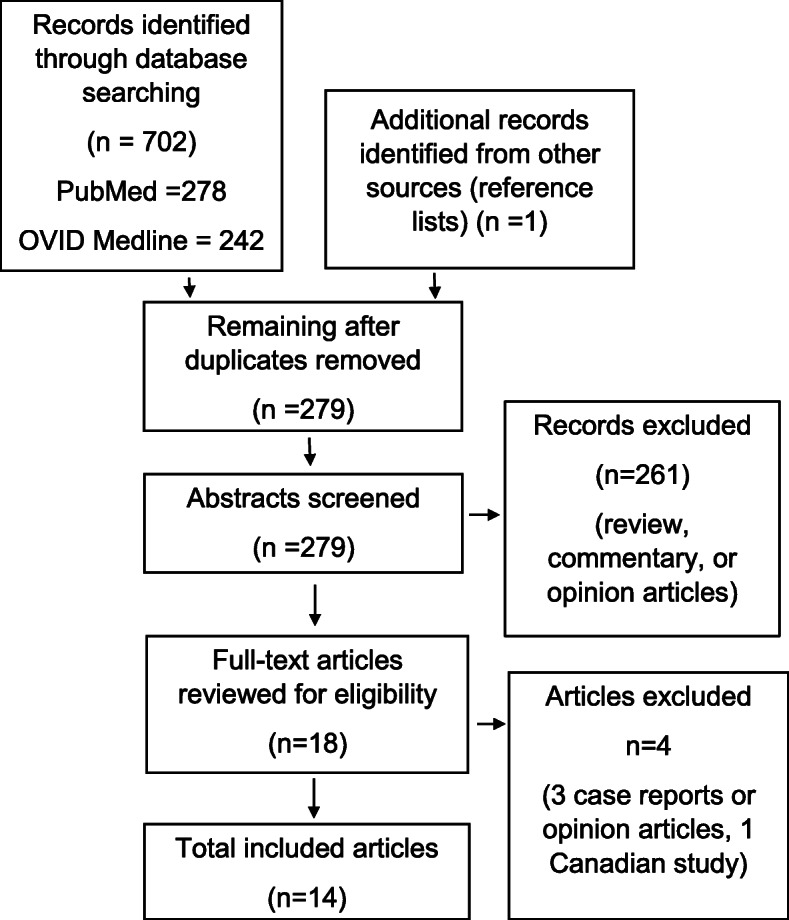
PRISMA Diagram

Studies identified in the search process were screened by title and abstract by two researchers (KA and NH) and selected for full review if the studies: 1) reported opioid-related health outcomes during the COVID-19 pandemic, 2) were written in English, and 3) were original, empirical research conducted during the COVID-19 pandemic. Reviews, opinion articles and commentaries were excluded. In addition, abstracts that reported on health outcomes not related to opioid use (i.e. COVID-disease mortality rates for PWUO) were excluded due to the focus on opioid-related health care delivery. Duplicates were removed across the databases. The screening process was refined prior to initiation by research team members (K.A., N.H.), and any protocol irregularities were discussed as an entire research team (K.A., NH, AG, MPM). Articles that were eligible for full text review were cross-checked by two additional reviewers, and disagreements were discussed until consensus was achieved. The search process is reflected in a PRISMA diagram (see Fig. 2).

### Data extraction and synthesis

Data from articles that were eligible for full text review were extracted into a table with the following headings: author, year of publication, study location, intervention (yes/no), study population, aim or purpose of the study, methodology, outcome measures, and key findings. The studies were reviewed with content analyses methodology [15] to categorize and interpret the findings across the varying methodologies of the articles. Full text articles were reviewed and coded by three reviewers (KA, AG, MPM), and agreement was achieved on categorization. The research team identified broad categories for the articles: service delivery, patient/provider experiences and patient outcomes. From these broad categories, empirical results of the reviewed studies allowed us to further refine these categories. While the categories are likely not exhaustive of all concepts associated with healthcare delivery to PWOU in the time of the COVID-19 pandemic, they provide a map or classification scheme to illustrate the types of evidence available.

## Results

The initial search was conducted in February 2021 and yielded 278 peer-reviewed articles. After the removal of duplicates, 47 review articles were immediately excluded; 210 opinion and commentaries were also excluded by reading the abstracts. Three articles were excluded because they did not report on health outcomes related to opioid use, but instead reported outcomes related to COVID-19 disease in people who use substances. One study [16] from Canada was excluded as as the policies governing OTP and OBOT vary greatly from the US and its inclusion would limit the generalizability of our review findings. Of the 18 articles reviewed in their full text version for inclusion, 14 articles met the inclusion criteria. Among the included studies, 79% (*n* = 11) were descriptive studies utilizing administrative database or medical chart review, one was a cross-sectional survey and the remaining two were qualitative studies. Articles were organized into three categories based on content: service delivery (*n* = 7), provider/patient experiences (*n* = 3), and patient outcomes (*n* = 4). Table 1 further describes the subthemes of the studies beneath the three broader themes.

**Table 1:** Categorization and descriptions of evidence

Theme/Authors/Country	Study Design(s)	Data Source(s)	Key findings
**Service delivery (*n*** **= 7, 47%)**			
*Prescriptions filled (n = 3)*			
McIlveen et al. [17] (U.S./Oregon)	Retrospectiv	Electronic medical record of outpatient OUD treatment centers in Oregon	In-person medication dosing visits declined 33%, and take home medication increased 97% as intended.
Jones et al. [18] (U.S./Nationwide)	Retrospective	IQVIA Total Patient Tracker Database	Buprenorphine was dispensed at an expected rate, but intramuscular naloxone was not.
Thornton et al. [19] (U.S./Texas)	Retrospective	Texas Prescription Monitoring Program dataset	Filling of daily buprenorphine prescriptions remained steady.
*EMS*^a^ *and ED*^b^ *utilization (n = 4)*			
Glober et al. [20] (U.S./Indiana)	Retrospective	EMS and coroner’s office record in Marion County, Indiana	47% increase in calls for overdose.
Holland et al. [21] (U.S./Nationwide)	Retrospective	Center for Disease Control and Prevention’s National Syndromic Surveillance Program	ED visits for reasons of opioid overdose were significantly higher during the pandemic compared to 2019 rates.
Slavova et al. [22] (U.S./Kentucky)	Retrospective	Kentucky State Ambulance Reporting System	71% increase in refusal of transport to a hospital following an overdose call to EMS. 50% increase in overdose calls involving death at the scene.
Weiner et al. [23] (U.S./Massachusetts)	Retrospective	Massachusetts Ambulance Trip Information System	After the stay-at-home order, most calls other than substance use dropped, but substance related EMS calls increased substantially.
**Provider and patient experiences (*n*** **= 3, 20%)**			
*Provider experiences (n = 2)*			
Caton et al. [24] (U.S./California)	Cross-sectional	338 clinicians from 57 primary care clinics in California	66% of clinics reported an easier time or an unchanged difference in retention and engagement of clients during the pandemic.
Collins et al. [25] (U.S./Rhode Island)	Qualitative	14 Emergency room healthcare providers	Barriers persist in connecting patients to outpatient treatment, including capacity and infrastructure to use telehealth and, COVID-test requirements as a barrier to accessing treatment.
*Patient experiences* (*n* = 1)			
Krawczyk et al. [26] (International)	Qualitative	300 posts of Reddit users related to COVID-19 and opioid used	Concerns over less in-person access to OUD treatment facilities, COVID-19 testing treatment requirements and exposure to COVID were reported as an influence on motivation to seek treatment.
**Patient outcomes (*n*** **= 5, 33%)**			
*Overdose (n = 3)*			
Khatri et al. [27] (U.S./Pennsylvania)	Retrospective	Philadelphia Dep Health Substance Use Dashboard and Medical Examiner’s Office Data	African Americans comprised 80% of overdose ED visits post-pandemic, compared to 63% pre-pandemic.
Ochalek et al. [28] (U.S./Virginia)	Retrospective	Electronic medical record of Virginia Commonwealth University	During the pandemic, 2.5 patients were seen per day in the emergency department with opioid overdose, compared to 1.4 patients per day pre-pandemic.
Rodda et al. [29] (U.S./California)	Retrospective	Electronic medical records and medical examiner records in San Francisco	Fatal overdoses significantly increased 60% in Non-Hispanic Black patients post-COVID restrictions, overdoses in Non-Hispanic White patients decreased.
*Retention in treatment (n = 1)*			
Tofighi et al. [30] (U.S./New York)	Retrospective	Bellevue Hospital (NYC) electronic medical record	56tg6

^a^Emergency Medical Services; ^b^Emergency Department

**Fig. 3:**
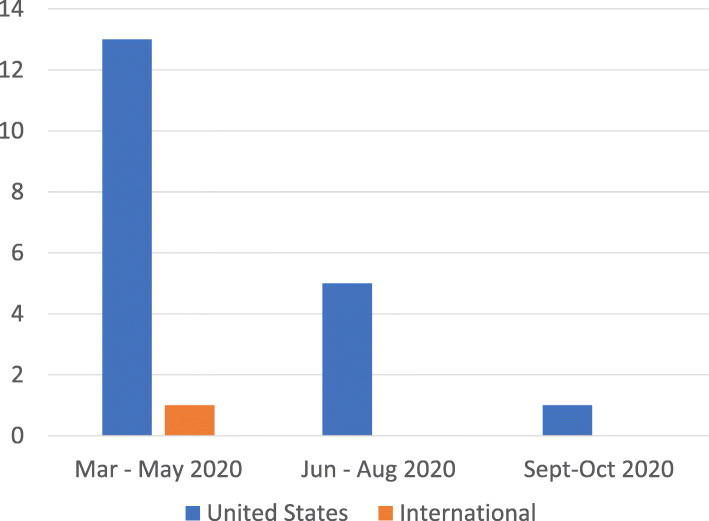
Distribution of Reported Data by Time Period and Country

Studies represented outcomes from a cross-section of the US healthcare system. The Eastern US (Massachusetts, New York City, New York, Philadelphia, Pennsylvania, Rhode Island) was most represented, with studies also conducted in the Southern (Virginia, Kentucky, Texas), Western (California and Oregon), and Midwestern US (Indiana). One qualitative study pulled data from the Internet site Reddit, and therefore drew from an international sample of Internet users. Studies primarily reported on data from the first 3 months of the pandemic, though one study reported on data through October 2020 (see Fig. 3).

### Service delivery

Seven studies (64%) examined service delivery using administrative datasets across different geographic regions in the United States analyzing pandemic changes in MOUD dispensing (*n* = 3) and ED/emergency medical services (EMS) utilization (*n* = 4). Following the federal guidance adjustments on March 16th, 2020, in-person medication visits at OTP for MOUD declined 33% and take-home medication (methadone or buprenorphine) increased 97% in Oregon. Telehealth use for MOUD initiation and decreased restrictions on take-home medication had its intended effect of reducing crowding in OTP clinics [17]. Using nationwide prescription tracking data, buprenorphine was dispensed nationally at an expected rate through OBOT or OTP, but intramuscular naloxone - an opioid reversal drug - was not [18]. Similarly, despite a significant decline in healthcare utilization in Texas, the level of daily buprenorphine prescriptions filled remained steady, again suggesting that telehealth visits were able to meet the medication prescribing needs of this population [19].

In Indiana, EMS providers recorded a 61% increase in administration of naloxone to reverse suspected opioid-related overdoses and a 47% increase in overdose-related calls [20]. There were no changes in frequencies of overdose based on race or zip code. In Kentucky, a 17% increase in calls to EMS for overdose was reported, as well as a 71% increase in patients refusing transport to the hospital following an overdose EMS call [22]. The study also reported a 50% increase in overdose calls involving a death at the scene. In Massachusetts, an initial decrease in all calls to EMS in March 2020, was followed by a subsequent increase in substance-related EMS calls by May 2020 [23]. The study also reported a high refusal rate of hospital transportation in Massachusetts. Nationwide, while all-cause weekly ED visits decreased sharply from March to May 2020, weekly ED visits for reasons of opioid overdose were significantly higher during this period when compared to March to May 2019 rates (*n* = 5502 vs 4168, *p* < .001).

### Provider and patient experiences

Three studies (27%) described perceived treatment access from provider and patient perspectives in a cross-sectional survey (*n* = 1) and qualitative (*n* = 2) studies. A survey of primary care practices in California (*n* = 57) found that the majority (91.2%) of OTP clinics adapted their practices in response to COVID-19 with the most common change being use of telehealth appointments [24]. Clinics also reported prescribing buprenorphine for longer durations since the onset of the pandemic and 67% of clinics reduced the frequency of urine drug screenings for established patients [24]. Over half of practices reported better retention and engagement with patients in care using the partial telehealth service model, including increased demand for counseling and behavioral health services. ED physicians reported difficulty referring patients to begin community OTP due to increased COVID-19 related restrictions in capacity and infrastructure [25]. Finally, patients reported uncertainty about accessing OTP due to possible exposure to COVID-19 infection and increased COVID-19 related restrictions in facilities [26]. Telehealth access to buprenorphine perceived by patients to increase access to treatment; the increase in take-home medication was, however, seen as risky by some patients, especially when they were in the early days of treatment [26].

### Patient outcomes

Four studies (36%) examined patient outcomes using either electronic health records or medical examiner data in the US during the pandemic; outcomes included retention in treatment (*n* = 1), and overdose rates (*n* = 3). In Philadelphia, fatal overdoses increased by 60% in Non-Hispanic Black individuals when comparing April to June 2020 to April to June 2019 data [27]. Virginia reported 80% of overdose visits to the ED were among Black patients during the COVID-19 pandemic, compared to 63% pre-pandemic [28]. In San Francisco, fatal overdoses in the ED nearly doubled during the pandemic compared to pre-pandemic rates [29]. This contrasted with a decrease in fatal overdoses among Non-Hispanic White patients post-COVID restrictions in San Francisco [29].

One study examined treatment retention for new patients (*n* = 78) entering OUD treatment with a buprenorphine-naloxone induction via telehealth in New York City during the pandemic. At 8 weeks, 42 patients remained in the program (53.8%), 21 were transitioned to a community treatment program (26.9%), 15 were lost to follow-up (19.2%), and none were terminated from care due to suspicions of diversion or misuse [30]. Telehealth was seen to be feasible, safe and as effective at retaining patients in treatment with buprenorphine as in-person visits.

## Discussion

Healthcare services for PWUO quickly shifted at the onset of the COVID-19 pandemic to accommodate the public health guidance set forth to reduce the spread of COVID-19 while also incorporating changes in federal and local policies for the administration of MOUD. In this scoping review, we found that healthcare utilization dropped quickly starting in March 2020, with the exception of care for opioid overdoses which sharply increased beginning in May 2020. Service gaps in accessing treatment during the pandemic due to capacity and infrastructure limits were reported by patients and providers. Disproportionate increases in overdose rates among Black patients were reported in two studies, with differences by race and gender not reported in most studies. This scoping review highlights the substantial unmet need for connection to OUD treatment during the pandemic as rates of overdose and fatality rose substantially, particularly for already marginalized populations. Overall, the evidence from this scoping review suggests that greater flexibility in the provision of MOUD was necessary and beneficial for PWUO and merits support post-pandemic.

During the beginning months of the pandemic, receipt of MOUD continued at usual rates in three large studies, demonstrating minimal disruption in providing treatment to patients through telehealth and take-at-home medication protocols. However, barriers persisted in connecting new patients to treatment with evidence from patients and providers citing capacity and infrastructure limits [25, 26]. This may have inadvertently exacerbated race/ethnic treatment disparities, as White PWUO were more likely to be engaged in services prior to the pandemic than BIPOC PWUO. Fears about going to hospitals and clinics during the pandemic may have prevented many PWUO from initiating treatment. However, there was also signs of hope as those who received a MOUD were more likely to complete their scheduled healthcare visits and to pick up their medications even while healthcare use overall declined. This suggests that these services were perceived as vital to those receiving them. As noted by experts previously, a crucial next step to meeting this service gap involves ensuring technology access and support for all groups of PWUO and ensuring low-threshold pathways for PWUO to access medication [31, 32].

The ED emerged as a critical point for potential patient engagement, as rates of patients presenting in the ED for overdose rose sharply during the pandemic. In response to this shift, a bill was passed in December 2020 (East MAT Act, H.R. 2281) that allowed for providers without an X-waiver to provide up to a 72-h supply of buprenorphine to patients in acute opiate withdrawal. Prior to this, patients were able to receive a 72-h supply if they returned to the ED for their daily dose. In addition, as of April 28, 2021, all DEA licensed providers are able to apply for an X-waiver without completing specific training as long as they are treating fewer than 30 active patients at a time, thus further increasing capacity for ED providers to serve as a site of initial patient engagement for initiating MAT [33].Incentivizing ED physicians to become authorized to provide buprenorphine or allowing longer term prescriptions for buprenorphine from the ED could be critical policy initiatives to ensure greater and faster entry into outpatient treatment [34].

Telehealth was described as an important service model to connect PWUO with buprenorphine treatment during the emergence of COVID-19 [30]. Prior to COVID-19, while the use of telehealth for substance use disorders had increased 20 fold from 2010 to 2017, it remained overall underutilized, as telehealth visits for substance use disorders only made up 1.4% of all telehealth visits in 2017 [35]. Telehealth waivers from the Centers for Medicare & Medicaid Services, expansion of telehealth services through Federally Qualified Health Centers and Rural Health Clinics and flexibility with provision of telehealth services through applications not fully compliant with HIPAA rules since COVID-19 have made it easier for health care providers to deliver telehealth services [36]. Additionally, it relaxed requirements for an in-person visit to initiate buprenorphine treatment, allowing for telehealth or telephone visits, which should be allowed to continue post-pandemic as a means of low-barrier access to care. However, challenges in connecting patients and especially marginalized populations such as PWUO remain including access to adequate technology and internet access, especially in persons living in transitional housing or homeless populations [31]. Future work should explore approaches to address barriers to telehealth uptake among PWUO.

As demonstrated in this review, overdose continues to be a growing consequence of opioid misuse, and racial disparities that existed pre-pandemic are intensifying. In June 2020, a Kaiser Family Foundation poll found that 13% of adults in the US reported increased substance use due to COVID-19 related stress, and 40% of adults reported symptoms of anxiety or depression, compared to 10% in June 2019 [37]. Previous pre-pandemic literature pointed to depression and anxiety as associated with overdose risk in general populations [38], and more prevalent in marginalized populations racial minorities [38, 39].

In addition, the effects of structural racism that were present prior to the pandemic were intensified as overdose disproportionately affected Black, Indigenous and People of Color (BIPOC). BIPOC are less likely than others to have access to healthcare in the US, and are more likely to be arrested and incarcerated instead of receiving treatment for drug-related issues [40]. White PWUO were more likely to be in treatment at the start of the pandemic, and therefore were more likely to maintain access to treatment. Increasing access to treatment for BIPOC could be accomplish through increasing options for entering treatment (ED, primary care, telehealth), through culturally appropriate public health campaigns, and increasing the diversity of the addiction workforce [41]. Due to the ongoing nature of the pandemic, the unintended consequences of the COVID-19 pandemic on PWUO must be further studied, especially for vulnerable subgroups, with a focus on developing actionable policy and clinical changes to improve healthcare delivery for this population.

### Gaps in the literature

A comprehensive assessment of the consequences of the pandemic on healthcare delivery for PWUO is not attainable with existing data. Most published data available to inform practice and policy report on the first 3 months of the pandemic (see Fig. 3) with only two studies quantifying outcomes into Fall 2020. It is possible that trends in overdose have further increased due to the long-term nature of societal changes related to the pandemic, though this knowledge is lacking to date.

Further, all studies occurred in the US, highlighting an important gap in the literature (see Fig. 3). The pandemic and OUD are both global crises, with waves occurring in synchronous patterns. Future research from developing countries is important to describe and understand the global impact of these overlapping epidemics. Finally, studies only described the dispensing and administration of buprenorphine and long-acting naltrexone. Methadone is the most dispensed MOUD, yet it is not represented in the evidence analyzed. The one study that reported attendance at OTP visits did not distinguish between administration of methadone or buprenorphine [17].

### Limitations

Our review is limited by the gaps in the literature, as outlined above. In addition, interpretation of findings is limited due to challenges with disentangling changes in healthcare delivery in the context of the COVID-19 pandemic due to the multiple confounding variables present in society across multiple levels (fear to engage in public, technology access gaps, loss of jobs, increased drug tainting, etc). Importantly, we do not make the conclusion that the policy changes to OUD treatment delivery caused the increases in ED visits or overdoses, rather we describe the evidence with the intention of highlighting trends and prompting future research. We also did not include articles involving COVID-19-related illness in PWUO as this has been reported elsewhere [11]. Although COVID-19 is reported to produce more serious disease to individual health for PWUO it was outside the scope of this review.

## Conclusion

In this scoping review, we found that despite changes in healthcare policy designed to increase access to MOUD for PWUO, overdose related calls and deaths continued to increase throughout the first months of the COVID-19 pandemic. Further, racial minority populations were disproportionately affected at higher rates by the intersection of COVID-19 and OUD. As the pandemic is ongoing, future research must focus on developing tailored interventions to reduce morbidity and mortality for PWUO, while addressing the needs of racial minorities.

## Data Availability

All data generated or analyzed during this study are included in the article, especially in Table 1.

## Abbreviations

US: United States
PWUO: People who use opioids
OUD: Opioid use disorder
MOUD: Medications for opioid use disorder
ED: Emergency Department
COVID-19: Coronavirus-19
SAMHSA: Substance Abuse and Mental Health Services Administration
EMS: Emergency Medical Services
PRISMA: Preferred Reporting Items for Systematic Reviews and Meta-Analyses
